# Comparative Dental Anatomy

**Published:** 1884-07

**Authors:** Morgan Adams

**Affiliations:** Sardis, Miss.


### Comparative Dental Anatomy.

BY MORGAN ADAMS, M. D., D. D. S., SARDIS, MISS.

Read before the West of Scotland Branch of the British Dental Association, Glasgow, Feb. 9th, 1884.

The incisor here presented is from the jaw of a common black bear which was slain in the Mississippi swamps. There is nothing particularly interesting in it except its great strength and its beautiful dense and polished enamel. It is well for us in our researches to turn aside from the human teeth when opportunity presents, and consider the qualities and differences in the teeth of wild animals and those of the domesticated, and compare them with the teeth of man. This is a fair specimen of the teeth of the black bear in its natural or wild state. In its formation and texture it is faultless, being strong enough to crush the bones of a man's arm, and its beautiful enamel looks as though it were just from the hands of the lapidary. These qualities are to some extent characteristic of most wild animals, those of the beaver being most notable. I, myself, have seen trees cut down nearly as large as a man's body by these little animals. Let us now notice briefly the difference in the teeth of domestic animals. It is well known that the teeth of the dog are often worn off or wasted away by erosion, and his usefulness diminished long before he dies of old age, and I have often seen their teeth covered with tartar, and in some cases not well developed. A house cat was noticed suffering great pain ; upon examination a diseased tooth was found to be the cause. It is also a well known fact that domesticated monkeys sometimes suffer from diseased teeth. I have never seen nor heard of any of these defects or troubles in the teeth of wild animals, and my opportunities for observation in this particular have been considerable. The difference in the teeth of the savage and the civilized man are well known to all who have given the subject the least thought. I have examined many skeletons of prehistoric races in America, and have never found caries, or evidence of any other disease in their teeth. I have had some opportunity of examining skeletons of the wild tribes now living in America. Caries are occasionally found in their teeth, but it is well known that they have far better teeth than men of civilized nations. Now we are told by investigators that bad teeth is one of the penalties of civilization, and they tell us the truth, but do they tell us the truth when they affirm that bad teeth are caused entirely from eating bread that has been deprived of a part of its nutritive principles? Compare this tooth of the wild bear with the teeth of the best fed civilized man, and note the difference in them. Savages and wild beasts feed upon raw food; this is given by some as the reason of their good teeth; but does this satisfy us? Does any thing as yet set forth fully satisfy us as to the cause of bad teeth? I answer no, but do not presume to offer anything myself. I only throw out these few hints with the hope that some one may think about them, and give us some light upon a subject that is of vital importance to mankind.

